# The Relationship Between Substance Use, Academic Performance and Self-Esteem Among Nursing Students

**DOI:** 10.1192/j.eurpsy.2026.10869

**Published:** 2026-07-31

**Authors:** A. Triki, A. Kchaou, N. Kotti, F. Dhouib, M. Hajjaji, K. J. Hammami

**Affiliations:** Occupational Health, Hedi Chaker University Hospital, Sfax, Tunisia

## Abstract

**Introduction:**

Substance use is an emerging concern among healthcare trainees, particularly nursing students, who face high academic and clinical stress while being expected to maintain their mental health and serve as role models for healthy behaviors.

**Objectives:**

To examine the associations between substance use, academic performance, and self-esteem among nursing students in Sfax, Tunisia.

**Methods:**

A cross-sectional study was conducted during the academic year 2024–2025 among all nursing students enrolled at the Higher Institute of Nursing Sciences of Sfax (N=266). Data collection included a sociodemographic questionnaire, patterns of tobacco, alcohol, and cannabis use, academic performance indicators, and the Rosenberg Self-Esteem Scale.

**Results:**

Substance use prevalence was: tobacco 16.2% (n=43), alcohol 3.4% (n=9), and cannabis 4.1% (n=11). Low academic performance was reported by 10.5% (n=28). The mean Rosenberg Self-Esteem score was 28.5 ± 3.7 (range: 14–40), and low self-esteem was found in 71.4% (n=190). Tobacco, alcohol, and cannabis use were all significantly associated with poorer academic performance (p<0.001, p=0.026, and p=0.016, respectively) and with lower self-esteem (p=0.004, p=0.018, and p=0.014, respectively).

**Conclusions:**

Substance use is significantly linked to both academic difficulties and reduced psychological well-being among nursing students. These findings call for comprehensive prevention and support programs that address substance use while promoting mental health and academic success in nursing education.

**Disclosure of Interest:**

None Declared

